# Protective Effects of Olive Leaf Extract on Acrolein-Exacerbated Myocardial Infarction via an Endoplasmic Reticulum Stress Pathway

**DOI:** 10.3390/ijms19020493

**Published:** 2018-02-07

**Authors:** Yuyu Xu, Lixing Wu, Aochang Chen, Chaoqi Xu, Qing Feng

**Affiliations:** 1 Department of Nutrition and Food Hygiene, Nanjing Medical University, Nanjing 211166, China; yuyuxuup@njmu.edu.cn (Y.X.); foxwlx@126.com (L.W.); xiaocc1992@njmu.edu.cn (A.C.); chaoqixu2015@163.com (C.X.); 2 Department of Cardiology, Jiangsu Province Hospital, The First Affiliated Hospital with Nanjing Medical University, Nanjing 210000, China

**Keywords:** acrolein, apoptosis, olive leaf extract, hydroxytyrosol, oleuropein

## Abstract

Many studies reported that air pollution particulate matter (PM) exposure was associated with myocardial infarction (MI). Acrolein representing the unsaturated aldehydes, the main component of PM, derives from the incomplete combustion of wood, plastic, fossil fuels and the main constitute of cigarette smoking. However, the effect of acrolein on MI remains not that clear. In the current study, the effect of acrolein-exacerbated MI was investigated. In vivo, male Sprague–Dawley rats received olive leaf extract (OLE) followed by acrolein, then isoprenaline (ISO) was received by subcutaneous injection to induce MI. Results showed that the expression levels of GRP78 and CHOP, two major components of endoplasmic reticulum (ER) stress were higher in the combination of acrolein and ISO than those in ISO treatment. The apoptosis marker, Bax, was also higher while the anti-apoptosis indicator, Bcl2 expression was lower both at protein and mRNA levels in the combination group. Also, the acrolein-protein adducts and myocardial pathological damage increased in the combination of acrolein and ISO relative to the ISO treatment. Besides, cardiac parameters, ejection fraction (EF) and fractional shortening (FS) were reduced more significantly when acrolein was added than in ISO treatment. Interestingly, all the changes were able to be ameliorated by OLE. Since hydroxytyrosol (HT) and oleuropein (OP) were the main components in OLE, we next investigated the effect of HT and OP on cardiomyocyte H9c2 cell apoptosis induced by acrolein through ER stress and Bax pathway. Results showed that GRP78, CHOP and Bax expression were upregulated, while Bcl2 expression was downregulated both at the protein and mRNA levels, when the H9c2 cells were treated with acrolein. In addition, pretreatment with HT can reverse the expression of GRP78, CHOP, Bax and Bcl2 on the protein and mRNA levels, while there was no effect of OP on the expression of GRP78 and CHOP on the mRNA levels. Overall, all these results demonstrated that OLE and the main components (HT and OP) could prevent the negative effects of acrolein on myocardium and cardiomyocytes.

## 1. Introduction

Cardiovascular diseases still remain the main cause of increased mortality worldwide, among which Ischemic heart disease and stroke are the most prevalent [1,2]. As to ischemic heart disease, myocardial infarction (MI) is an acute status of unbalance between decreased coronary flow and the demand of the myocardium. A growing number of studies demonstrate the significant association between myocardial infarction and air pollution exposure [3,4,5,6]. For instance, epidemiological studies showed that short-term exposure of particulate matter (PM) exacerbated myocardial infarction. In other words, the patients suffering from ischemic heart diseases were more susceptible to particulate matter exposure [3,7]. Furthermore, carbonyl compounds (aldehydes and ketones) proved to be the key molecular components of the water soluble part of PM [8]. Particularly, acrolein, an ubiquitously unsaturated aldehyde was generated during the incomplete combustion of wood, plastic, fossil fuels and the main constitute of cigarette smoking [9]. In addition, it can be discovered in carbohydrate-rich food that is cooked at high temperature as a result of the Maillard reaction [10]. The average concentration of acrolein was 2.3 ± 1.0 μg/m^3^ at a heartland of Beijing site from June to October in 2008 [11]. Also, acrolein emitted per cigarette is 10–500 µg [12,13]. Acrolein readily forms covalent adducts with proteins and DNA due to being a strong electrophile, which result in cytotoxicity that induces various diseases including cardiovascular diseases [14]. Recent studies show that exposure to acrolein increased cardiovascular disease risk [15,16,17]. However, the association between acrolein and myocardial infarction has not yet been elaborated clearly.

OLEs from olive leaves and branches are rich in polyphenols, of which oleuropein (OP) and hydroxytyrosol (HT) are the major phytochemical components that play important roles in anti-atherosclerotic, cardioprotective and hypoglycemic aspects [18]. Likewise, OP and HT are indispensable ingredients in the well-known Mediterranean diet that is associated with a low incidence rate of cardiovascular diseases [19,20,21]. Recent studies show that the protective effects of olive leaf extract (OP and HT) on cardiovascular diseases, but the explanation of mechanisms are severely insufficient [22,23,24].

In the current study, in vivo, we investigated acrolein exacerbated myocardial infarction that was induced by ISO in rats. Besides, the oral administration of OLE can reverse heart damage to some extent. In vitro, we explored the protective effects of HT and OP against the acrolein-inducing cardiomyocyte H9c2 cell apoptosis through ER stress and Bcl2/Bax pathways.

## 2. Results

### 2.1. Effect of OLE on Aggravated Myocardial Injury Induced by Acrolein

**Figure 1:**
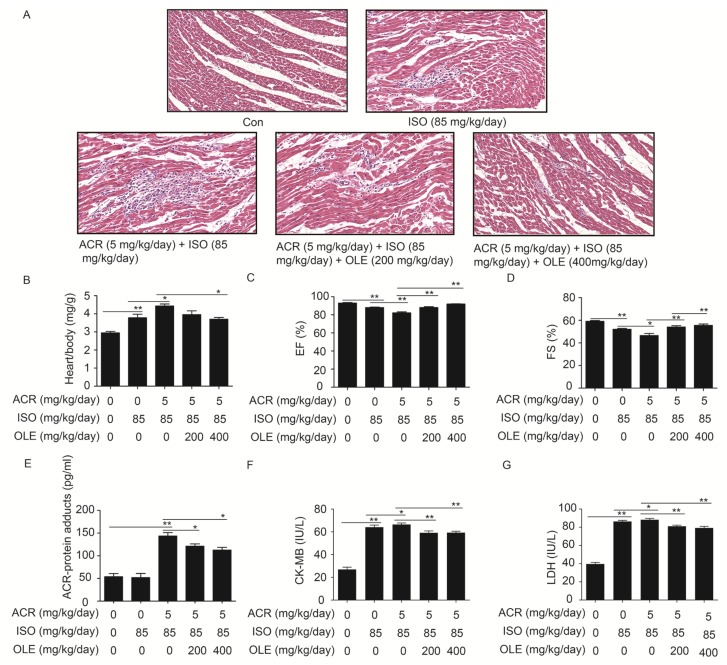
Effect of olive leaf extract (OLE) on aggravated myocardial injury induced by acrolein in rats. (**A**) Representative photographs of heart sections stained with haematoxylin and eosin (200×) in different groups; (**B**) quantitative analysis of the heart weight over body weight ratio in different groups; (**C**,**D**) Quantitative analysis of left ventricular FS (%) and EF (%), as tested by echocardiography; (**E**) the levels of acrolein-protein adducts in different groups, as measured by ELISA; (**F**,**G**) the levels of myocardial enzyme CK-MB and LDH in different groups, as measured by ELISA. * *p* < 0.05, ** *p* < 0.01.

Following the HE staining, there were no apparent changes in the control group; the structure of the myocardium was normal and cells were well-arranged. In the ISO treatment group, the structure of myocardium was disorderly and myocardial cells presented widespread edema. Besides, distinct infiltration of inflammatory cells were observed. In the combination of acrolein and ISO treatment group, the tissue was suffering more serious damage relative to the ISO group. Cell gaps grew bigger, the cell nucleus was not clear and more severe infiltration of inflammatory cells was observed. In the pretreatment with OLE (200 mg/kg/day, 400 mg/kg/day) groups, the myocardial tissue damage was reduced, and infiltration of inflammatory cells was decreased (Figure 1A). As shown in Figure 1B, the heart weight over body weight ratio in the combination of ISO and arolein treatment group was higher than that in ISO treatment group. However, the accretion can be reversed by OLE to some extent. Results from echocardiography showed that the parameters of cardiac systolic function, ejection fraction (EF) and fractional shortening (FS), were lower in the combination of ISO and acrolein treatment group than in the ISO treatment group. Also, OLE pretreatment reversed the value of EF and FS (Figure 1C,D). Furthermore, the acrolein-protein adduct, myocardial enzyme CK-MB and LDH were detected from the collected serum. It is obvious that the concentrations of acrolein-protein adducts, CK-MB and LDH were highest in the combination of ISO and acrolein treatment group, and the increase was reversed by OLE pretreatment (Figure 1E). All the results demonstrated that acrolein worsened the myocardial injury induced by ISO, and that OLE can attenuate the damage significantly.

### 2.2. Effect of OLE on Myocardial Apoptosisand Infiltration of Macrophagesinduced by Acrolein

**Figure 2:**
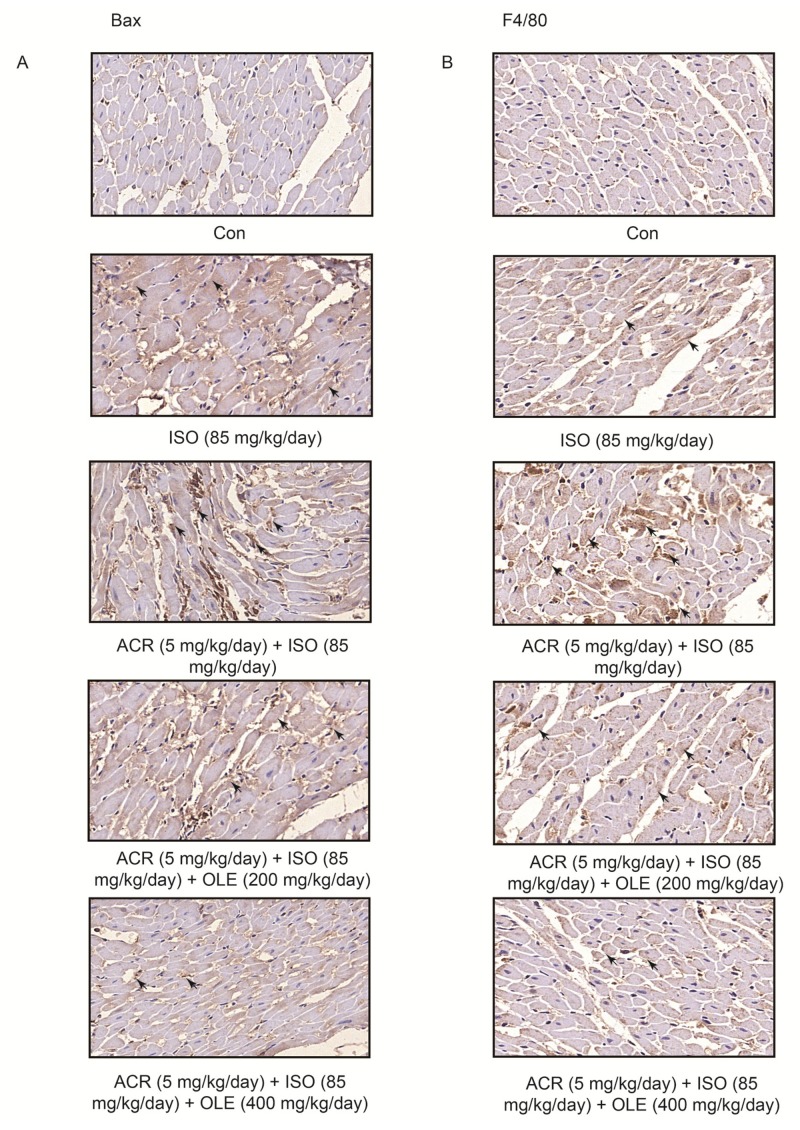
Effect of OLE on myocardial apoptosis and infiltration of macrophages induced by acrolein. (**A**,**B**) Representative photographs of heart sections with immunohistochemical staining of Bax and F4/80 (400×) in different groups.

To explore the effect of OLE on myocardial apoptosis, we performed immunohistochemical staining of Bax. The expression of Bax in the combination of ISO and arolein treatment group was strongly positive, and the distribution of Bax was disordered. As expected, the increased expression of Bax was reversed by OLE (200 mg/kg/day, 400 mg/kg/day) in a dose-dependent manner (Figure 2A). To explore the infiltration of macrophages, immunohistochemical staining of F4/80 was conducted. We observed massive macrophage cell infiltration and overexpression of F4/80 in the combination of ISO and arolein treatment group. Also, OLE (200 mg/kg/day, 400 mg/kg/day) can attenuate the infiltration of macrophages in a dose dependent manner, which has been marked by arrows (Figure 2B).

### 2.3. Effect of OLE on Myocardium Protection through the Pathway of ERS and Bcl2/Bax

**Figure 3:**
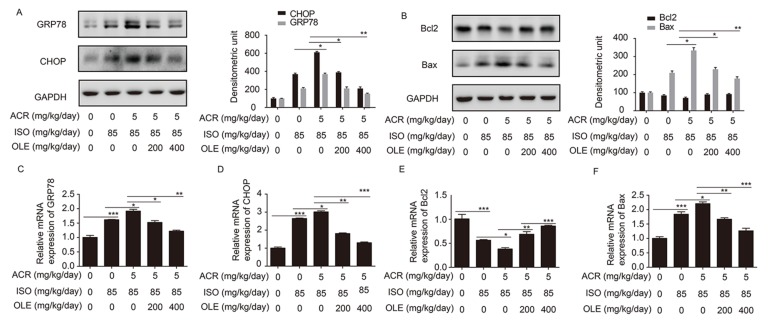
Effect of OLE on myocardium protection through the pathway of ERS, Bcl2/Bax in rats. (**A**,**B**) The protein expression of GRP78, CHOP, Bcl2 and Bax in different groups, as detected by Western blotting; (**C**–**F**) The mRNA expression of GRP78, CHOP, Bcl2 and Bax in different groups, as detected by qRT-PCR. * *p* < 0.05, ** *p* < 0.01 and *** *p* < 0.001.

To further confirm the mechanism on acrolein-inducing the increase in myocardial injury, the critical molecules GRP78 and CHOP in the ER stress pathway and apoptosis-related molecules Bcl2/Bax were examined. The expression of GRP78, CHOP and Bax at the protein and mRNA levels were increased more obviously in the co-treatment of acrolein and ISO rats than that in ISO treatment rats in comparison with controls. Meanwhile, Bcl2 was downregulated much more remarkably in the co-treatment of acrolein and ISO group than in the ISO treatment group. All the altered expression of GRP78, chop, Bax and Bcl2 at the protein and mRNA levels were reversed dramatically by pretreatment with OLE (200 mg/kg/day, 400 kg/kg/day) (Figure 3A–F). These data showed that acrolein exacerbated myoardialinjury induced by ISO through upregulation of GRP78, chop, Bax while showing the downregulation of Bcl2, which resulted in myocardial cell apoptosis. However, all the changes were ameliorated by OLE apparently.

### 2.4. HT and OP Reversed the H9c2 Cells Apoptosis Induced by Acrolein

**Figure 4:**
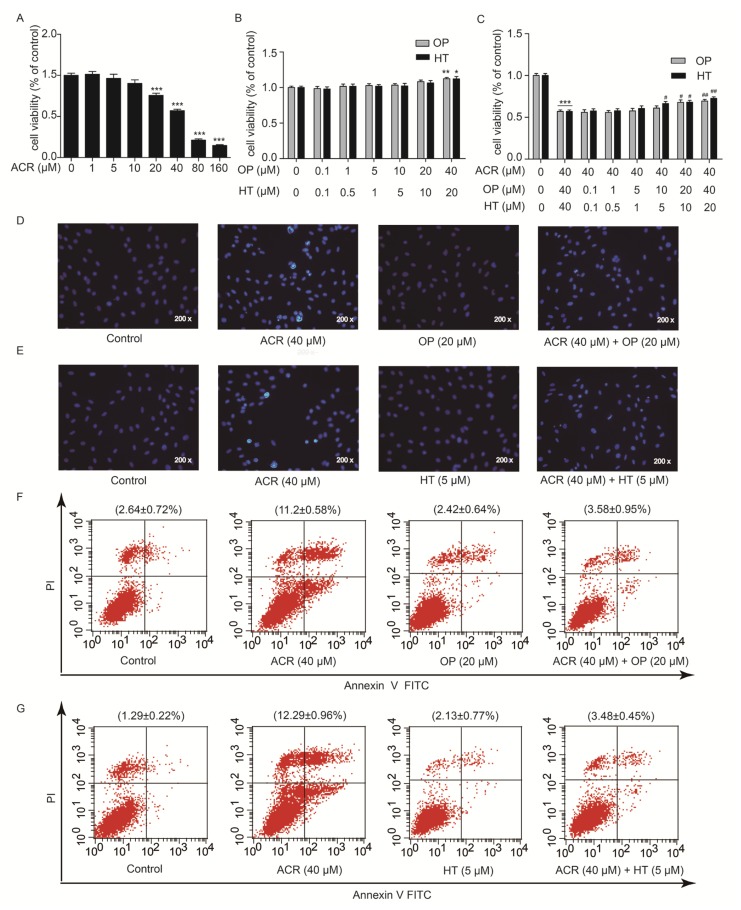
Hydroxytyrosol (HT) and OP reversed the H9c2 cells apoptosis induced by acrolein. (**A**) The cell viability of H9c2 after being treated with acrolein (0, 1, 5, 10, 20, 40, 80, 160 µM) for 12 h, as measured by MTT assay; (**B**) The cell viability of H9c2 after treatment with HT (0, 0.1, 0.5, 1, 5, 10, 20 µM) or OP (0, 0.1, 1, 5, 10, 20, 40 µM) for 24 h, as measured by MTT assay; (**C**) The cell viability of H9c2 after pretreatment with HT (0, 0.1, 0.5, 1, 5, 10, 20 µM) or OP (0, 0.1, 1, 5, 10, 20, 40 µM) for 24 h, and combination with acrolein (40 µM) for another 12 h, as measured by MTT assay; (**D**–**G**) the apoptosis rate of H9c2 after pretreatment with HT (5 µM) and OP (20 µM) for 24 h, and the combination with acrolein (40 µM) for another 12 h, as detected by Hochest 33258 staining (original magnification is 200×) and Annexin-V FITC/PI staining flow cytometry. Statistical differences to the controls were shown as * *p* < 0.05, ** *p* < 0.01 and *** *p* < 0.001. Statistical differences to the grouptreated with acrolein (40 µM) solely were shown as ^#^ *p* < 0.05 and ^##^ *p* < 0.01.

To explore which component of OLE plays an important role in protection against acrolein-induced myocardial injury, the main components of OLE, HT and OP were applied to cardiomyocyte H9c2 cells. MTT assays, Hoechst 33258 staining and flow cytometry were performed in vitro to detect the cytotoxicity of acrolein and whether HT and OP could prevent cytotoxicity in H9c2 cells exposed to acrolein. As shown in Figure 4A, the reduction in cell viability was remarkable in a dose-dependent manner, when the H9c2 cells were treated with acrolein at the indicated concentrations for 12 h. The IC_50_ was 39.60 µM (95% CI (33.88 to 46.29)). There was no change observed in cell viability, when the H9c2 cells were exposed to HT and OP at the indicated concentrations for 24 h (Figure 4B). In addition, H9c2 cells were pretreated with HT and OP at the indicated concentrations for 24 h followed by incubation with acrolein (40 µM) for another 12 h. The result from Figure 4C showed that HT and OP increased cell viability which was decreased by acrolein in a dose-dependent manner. When H9c2 cells were pretreated with OP (20 µM) and HT (5 µM) for 24 h, followed by incubation with acrolein (40 µM) for another 12 h, Hoechst 33258 staining showed that OP and HT was able to protect H9c2 cells from acrolein-induced apoptosis, as shown in Figure 4D,E. Besides, Annexin-V FITC/PI staining also showed that OP and HT could ameliorate acrolein-induced H9c2 cell apoptosis, which was analyzed by flow cytometry (Figure 4F,G). The presented data indicated that HT reversed the H9c2 cell apoptosis induced by acrolein.

### 2.5. Acrolein Altered the Expression of GRP78, CHOP, Bcl2 and Bax in H9c2 Cells

**Figure 5:**
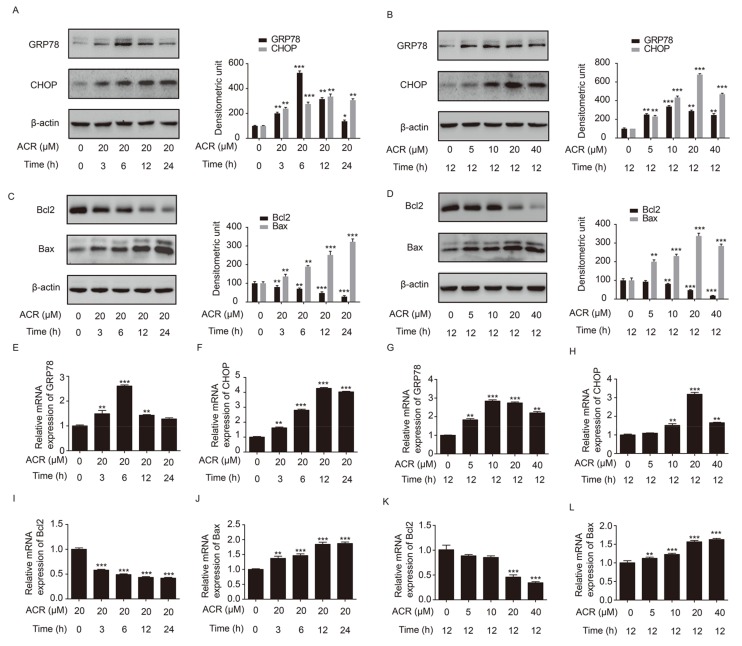
Acrolein altered the expression of GRP78, CHOP, Bcl2 and Bax in H9c2 cells. (**A**,**C**,**E**,**F**,**H**,**I**) The expression of GRP78, CHOP, Bcl2 and Bax at the protein and mRNA levels after H9c2 cells were treated with acrolein (20 µM) for the indicated time points (0, 3, 6, 12, 24 h), as measured by Western blotting and qRT-PCR; (**B**,**D**,**G**,**H**,**J**,**K**) The expression of GRP78, CHOP, Bcl2 and Bax at the protein and mRNA levels after the H9c2 cells were treated with acrolein (0, 5, 10, 20, 40 µM) for 12 h, as measured by Western blotting and qRT-PCR. * *p* < 0.05, ** *p* < 0.01 and *** *p* < 0.001.

To determine whether acrolein induced H9c2 cell apoptosis through the ER stress pathway and the mitochondrial pathway of apoptosis, key molecules, GRP78 and CHOP, of the ER stress pathway as well as Bcl2 and Bax from the Bcl-2 family of proteins were explored. The result showed that the expression of GRP78 and CHOP were increased after H9c2 cells were treated with acrolein (20 µM) for the indicated time points. Moreover, the level of GRP78 reached the peak at 6 h, then gradually returned to relatively lower levels. In addition, the level of CHOP reached the highest at 12 h, and was then reduced at 24 h (Figure 5A). As shown in Figure 5B, the expression of GRP78 and CHOP was increased after H9c2 cells were treated with acrolein at indicated concentrations for 12 h. In addition, it is obvious that the level of CHOP increased in a dose-dependent manner from 0 to 20 µM and decreased at 40 µM of acrolein. Furthermore, the expression of Bcl2 or Bax was decreased or increased in a time-dependent manner, when H9c2 cells were treated with acrolein (20 µM) for the indicated time points (Figure 5C). As shown in Figure 5D, the expression of Bcl2 or Bax was decreased or increased in a dose-dependent manner, when H9c2 cells were treated with acrolein at the indicated concentrations for 12 h. As expected, the mRNA expression of GRP78, CHOP, Bcl2 and Bax was consistent with the protein level, respectively, in the two kinds of treatments (Figure 5E–K). Therefore, the pathway of ER stress and mitochondrial pathway of apoptosis were involved in the process of acrolein-induced H9c2 cell apoptosis.

### 2.6. HT and OP Attenuated the Altered Expression of GRP78, CHOP, Bcl2 and Bax Induced by Acrolein in H9c2 Cells

**Figure 6:**
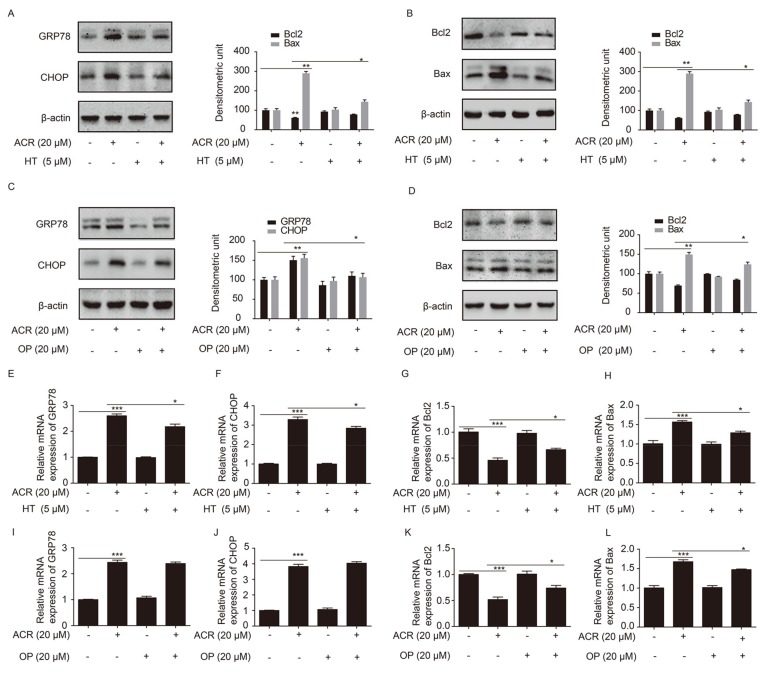
HT and OP attenuated the altered expression of GRP78, CHOP, Bcl2 and Bax induced by acrolein in H9c2 cells. (**A**,**B**,**E**–**H**) The expression of GRP78, CHOP, Bcl2 and Bax at the protein and mRNA levels after H9c2 cells were pretreated with HT (5 µM) for 24 h, and the combination with acrolein (20 µM) for 12 h, as measured by Western blotting and qRT-PCR. (**C**,**D**,**I**–**L**) The expression of GRP78, CHOP, Bcl2 and Bax at the protein and mRNA levels after H9c2 cells were pretreated with OP (20 µM) for 24 h, and the combination with acrolein (20 µM) for 12 h, as measured by Western blotting and qRT-PCR. * *p* < 0.05, ** *p* < 0.01 and *** *p* < 0.001.

To investigate whether HT and OP prevented H9c2 cell apoptosis through the ER stress pathway, the expression levels of GRP78, CHOP, and the the apoptosis makers Bcl2 and Bax were detected. As shown in Figure 6A,B, the levels of GRP78, CHOP and Bax were upregulated significantly when H9c2 cells were treated with acrolein (20 µM) for 12 h. At the same time, Bcl2 expression was obviously reduced. However, the change of GRP78, CHOP, Bcl2 and Bax was reversed when H9c2 cells were pretreated with HT (5 µM) for 24 h, followed by acrolein (20 µM) treatment for 12 h. Consistent with the protein expression, the altered expression of GRP78, CHOP, Bcl2 and Bax at the mRNA level due to acrolein was reversed by HT (Figure 6E–H). Besides, HT was replaced by OP (20 µM) to treat the cells, and other treatments were the same as the HT treatment group. As shown in Figure 6C,D, the altered expression of GRP78, CHOP, Bcl2 and Bax was alleviated by OP. In addition, the mRNA expression of Bcl2 and Bax is consistent with the protein expression (Figure 6K,L). However, the mRNA expression of GRP78 and CHOP was not reversed by OP (Figure 6I,J), which is necessary to explore further. 

## 3. Discussion

In the current study, we evaluated the effect of acrolein on exacerbating MI induced by ISO and the protective effect of OLE on myocardium from myocardialinjury in vivo. Also, we found that the preventive effect of HT and OP on cardiomyocyte H9c2 cytotoxicity generated by acrolein in vitro through ER stress and Bcl2/Bax apoptosis pathways. To our knowledge, this is first report that shows that OLE and the main components (HT and OP) regulated the ER stress pathway in H9c2 cells and myoardial injury rats against acrolein. In addition, our results supported the notion that acrolein and related aldehyde consumption increased the myocardial sensitivity to ischemia [17]. Therefore, our data demonstrated that OLE with abundant polyphenols and the main components (HT and OP) were helpful in the prevention and treatment of myoardial injury from air pollution exposure, such as the unsaturated aldehyde acrolein. ER is a significantly specialized organelle that consists of a huge membranous network, which exerts a vital role in directing synthesis and folding of proteins, as well as calcium homeostasis and phospholipid synthesis in eukaryotic cells [25,26,27]. However, once stimuli from internal and external environments disrupt the balance of protein synthesis and folding, misfolded and unfolded proteins are accumulated in the ER lumen, named as ER stress, which activates the unfolded protein response (UPR) to remedy the unbalance of the ER function. Three branches of ER stress transducers are involved including inositol-requiring protein 1 (IRE1), protein kinase ER-like kinase (PERK) and activating transcription factor 6 (ATF6) [28]. Under normal conditions, the ER chaperone 78-KD glucose-regulated protein (GRP78) was bound to the three ER stress transducers (IRE1, PERK and ATF6). When the accumulation of misfolded and unfolded protein triggers ER stress, GRP78 will be dissociated from the three ER stress sensors, resulting in activation of these molecules, which can ameliorate cell damage from dysfunction and promote cell survival [25,29]. However, prolonged UPR is able to elicit cell apoptosis that is regulated by C/EBP-homologous protein (CHOP, also named as GADD153), the specific transcription factor of ERS, which mediates cell apoptosis through many molecular pathways, such as the suppression of anti-apoptotic protein Bcl2, activation of caspase-12 and c-JUN NH2-terminal kinase [30,31,32]. It is reported that ER stress is associated with the pathogenesis of cardiovascular diseases including myocardial infarction, hypertrophy and heart failure [33]. There is continuous hypoxia and ischemia in myocardial infarct area, which gives rise to multiple folding enzymes located in ER lumen inactivation that induces misfolded and unfolded protein accumulation and ERS, also, excessive ERS initiates cell apoptosis [34,35]. Moreover, Thuerauf et al. demonstrated that UPR was activated in cardiomyocytes under hypoxia and the mouse model of myocardial infarction [36]. Besides, a previous study has showed that the expression of CHOP was significantly increased in the samples from heart failure patients and CHOP-deficient mice presented cardiac protective effects relative to wild-type mice, which verified that CHOP mediated the initiation of cell apoptosis [37]. Furthermore, the critical molecule of ER stress, GRP78, as the central area of protein folding in ER lumen, expression is upregulated to attenuate the ER stress through binding misfolded protein [38]. Therefore, the major molecules GRP78 and CHOP of the ER stress pathway, as well as apoptosis-related Bcl2/Bax were selected to elaborate the mechanism of ER stress on OLE against acrolein-worsening MI in rats and the antagonism of HT against acrolein in cardiomyocytes H9c2 cells. The results from this study showed that the expression of GRP78, CHOP and Bax were upregulated with downregulation of Bcl2 in MI rats induced by ISO, in addition, the combination of acrolein and ISO contributed to the much higher expression of GRP78, CHOP and Bax, as well as much lower Bcl2 in rats.

Apart from inefficient combustion of fossil fuels, acrolein was also discovered in endogenous lipid peroxidation [10,15]. The World Health Organization (WHO) working group estimated that the tolerable daily intake (TDI) of acrolein is 7.5 µg/kg body weight [10]. Also, the recommended maximum dose of acrolein is 65 µg/L, but this limit is usually exceeded [39,40]. The daily consumption of acrolein and related unsaturated aldehydes are estimated to be 5 mg/kg [17]. Hence, we selected 5 mg/kg concentration of acrolein representing the unsaturated aldehydes exposure in this study. A previous study showed that acrolein-protein adduct formation led to contractile depression [41]. In our results, the level of fractional shortening acrolein-protein adducts from acrolein gavage were high, but the acrolein-protein was decreased when the rats were treated with OLE by gavage for a month. In addition, cardiac parameters, EF and FS, were lower due to acrolein gavage relative to ISO treatment, while OLE reversed the low EF and FS, also, HT attenuated the cytotoxic effect of acrolein in cardiomyocyte H9c2 cells. However, various metabolic pathways are involved in the body, of which the formation of 3-hydroxypropylmercapturic acid (3-HPMA) from conjugation with glutathione (GSH) represents 60–70% of total acrolein metabolism excreted in urine [15]. A recent study demonstrated that glutathione S-transferase P (GSTP)-null mice were more sensitive to ischemia-reperfusion, which prevented the conjugation of acrolein and GSH [42]. Interestingly, OLE and the main component HT proved to have antioxidant effects [43,44,45]. OLE treatment has improved the hepatic GSH level in aged rats [46]. In our report, OLE treatment ameliorated acrolein-exaggerating myoardial injury may partly result from OLE restoring the level of GSH that bound acrolein, which attenuated ER stress. It is well known that OLE has high content of polyphenols including OP, HT and other flavonoids [47]. Many studies have reported that OLE and the polyphenol components OP and HT could exert cardioprotective effects, anticancer capacity and antidiabetic potential [48,49,50]. Our results support the cardioprotective effects of OLE and the main components (HT and OP) in vivo and in vitro. However, there was no effect of OP on the mRNA expression of GRP78 and CHOP. Given that, the role of OP may need further exploration. Besides, the difference in molecular structure between OP and HT may be the other reason to explain it [51]. Furthermore, this study would be improved and more convincing if clinical and epidemiological studies were added.

In summary, our data demonstrated that OLE ameliorated the acrolein-exaggerated myoardial injury and the main components (HT and OP) attenuated the cytotoxicity of acrolein in cardiomyocyte H9c2 cells through ER stress and Bcl2/Bax pathways. With the deteriorating air quality and the increase of PM 2.5 values, many people are inevitably exposed to acrolein and related unsaturated aldehydes, which have negative effect on human health. Therefore, it will be helpful to prevent and treat MI if OLE and the main components are supplemented reasonably.

## 4. Methods and Materials

### 4.1. Cell Culture and Reagents

H9c2 cells were purchased from the Cell Bank of Shanghai Institute of Biochemistry and Cell Biology (Shanghai, China). H9c2 cells were cultured in Dulbecco’s Modified Eagle Medium (DMEM, Gibco, Grand Island, NY, USA) supplemented with 10% fetal bovine serum (Gibco, Grand Island, NY, USA) and 1% penicillin-streptomycin mixed solution (Beyotime, Shanghai, China). The cells were maintained at 37 °C in a humidified 5% CO_2_ atmosphere. Acrolein (95%) was obtained from Sinopharm Chemical Reagent Company (Nanjing, China). Hydroxytyrosol (98%) was purchased from Aladdin Biological Reagent Company (Shanghai, China). Oleuropein (95%) was purchased from Solarbio Science and Technology Company (Beijing, China). Olive leaf extract (25%) was provided from Sinolife United Biotech Company (Nanjing, China). ISO was purchased from Yi Feixue Bio (Nanjing, China) for inducing MI in rat.

### 4.2. MTT Assay

H9c2 cells were seeded into 96-well plates at a density of 4000 per well with 200 µL medium under an environment of 37 °C, 5% CO_2_ and incubated overnight. After indicated treatments, methyl thiazol tetrazolium bromide (MTT, Amresco, OH, USA) solution (20 µL, 5 mg/mL, dissolved with PBS) was added to each well and the cells were incubated at 37 °C, 5% CO_2_ for 4 h. Then, dimethyl sulfoxide (DMSO, 150 µL) was added to each well to dissolve the formed formazan crystals at room temperature for 15 min after removing the medium. The solution was read at the absorbance of 490 nm with a microplate reader (Tecan/Infinite M200, Mannedorf, Switzerland).

### 4.3. Western Blot Analysis

H9c2 cells and cardiac tissue were lysed with RIPA including PMSF (Phenylmethanesulfonyl fluoride, Beyotime, Shanghai, China). The concentration of protein was checked by BCA Protein Assay Kit (Beyotime, Shanghai, China). Subsequently, the protein was separated with sodium dodecyl sulfate-polyacrylamide gel electrophoresis (SDS-PAGE) and transferred to polyvinylidene difluoride (PVDF) membranes (Millipore, Billerica, MA, USA). The primary antibodies included: anti-GRP78 (1:1000, proteintech, Wuhan, China), anti-CHOP and anti-F4/80 (1:1000, Cell Signaling Technology, Danvers, MA, USA), anti-Bcl2 and anti-Bax (1:500, Santa Cruz Biotechnology, Dallas, TX, USA), anti-β actin (1:1000, BOSTER, Wuhan, China), anti-GAPDH (1:1000, Beyotime, Shanghai, China). Secondary antibodies were: HRP-Conjugated AffiniPure Goat Anti-Rabbit IgG and HRP-Conjugated AffiniPure Goat Anti-Mouse IgG (1:2000, ZSGB-BIO, Beijing, China). Immunoreactive proteins were visualized using ECL Western blot detection reagents (Cell Signaling Technology, USA).

### 4.4. Real-Time Polymerase Chain Reaction (qRT-PCR)

Total RNA from cells and cardiac tissue was collected with RNAiso Plus (TaKaRaBio Technology, Dalian, China), following the manufacturer’s protocol. Reverse transcription was performed using the PrimeScriptTM RT Master Mix (TakaRaBio Technology, Dalian, China) and the qRT-PCR was performed using SYBR^®^ Premix EX TaqTM II (TakaRaBio Technology, Dalian, China) with the Applied Biosystems, 7900 Real Time PCR System (Applied Biosystems, Foster City, CA, USA). The primers were as follows: GRP78: (Forward—5′-TCAGCCCACCGTAACAATCAAGG-3′, Reverse—5′-CTTCCTCAGCAAACTTCTCGGCG-3′); chop: (Forward—5′-GCACCTCCCAAAGCCCTCGC-3′, Reverse—5′-CCGTTTCCTAGTTCTTCCTT-3′); GAPDH: (Forward—5′-CAAGGTCATCCATGACAACTTTG-3′, Reverse—5′-GTCCACCACCCTGTTGCTGTAG-3′); Bcl2: (Forward—5′-AGCCTGAGAGCAACCGAAC-3′, Reverse—5′-AGCGACGAGAGAAGTCATCC-3′); Bax: (Forward—5′-TTGCTACAGGGTTTCATCCAG-3′, Reverse—5′-TGTTGTTGTCCAGTTCATCG-3′). Expression of mRNA was calculated using the 2^−ΔΔCt^ method and normalization with GAPDH in cells, and the level of mRNA was calculated with 2^−ΔCt^ method in cardiac tissue.

### 4.5. Animals and Experimental Protocol

This animal experiment was performed in line with the recommendations in the Guide for the Care and Use of Laboratory Animals of the National Institutes of Health of China. The protocol was approved by the Institutional Animal Care and Use Committee of the Jiangsu Province Institute of Traditional Chinese Medicine. All male Sprague–Dawley rats weighing 200 ± 20 g were purchased from Shanghai Silaike Laboratory Animal Ltd. (Shanghai, China) and housed in the Animal Center of Jiangsu Province Institute of Traditional Chinese Medicine under the controlled conditions (temperature of 22 ±  1 °C, a 12-h light/12-h dark cycle and humidity of 55 ± 5%) acclimatized to the laboratory conditions for a week before starting experiment. Rats were randomized into five groups (*n* = 6): group (1), normal control rats received *dd*H_2_O by gavage for a month before subcutaneous injection of physiological saline for two successive days; group (2), ISO control rats received *dd*H_2_O by oral gavage for a month before subcutaneous injection of ISO (85 mg/kg/day) for two successive days; group (3): acrolein + ISO rats received *dd*H_2_O by gavage for 28 days, then the rats received acrolein (5 mg/kg/day) by gavage for two days before subcutaneous injection of ISO (85 mg/kg/day) for two successive days; group (4) and (5), rats received OLE at 200 mg/kg/day and 400 mg/kg/day for 28 days, then the rats received acrolein (5 mg/kg/day) by gavage for two days before subcutaneous injection of ISO (85 mg/kg/day) for two successive days. The animals were killed, and the blood and tissue samples were taken after injection of ISO for 48 h. Many studies reported that ISO (85 mg/kg/day) was used to induce MI [52,53,54]. Besides, ISO was detected at the different concentrations (5 mg/kg/day, 40 mg/kg/day, 85 mg/kg/day) to induce MI in our pilot experiment and the results indicated that ISO at 85 mg/kg/day presented the clearest effects on inducing MI. Wang et al. reported that consumption of unsaturated aldehydes can reach about 5 mg/kg/day, of which acrolein was selected to investigate the effect on MI [17]. The concentration of OLE referred to recent studies [55,56].

### 4.6. Histology

At the end of experimental period, all animals were killed and the hearts were harvested and fixed in 10% formalin solution for 24–48 h. After dehydration, tissues were embedded in paraffin. Sections were of the thickness of 5 µM that were stained with hematoxylin-eosin and antibody Bax and F4/80. The images were analyzed by Pannoramic Viewer after scanning.

### 4.7. Echocardiography

After anesthetizing with 10% chloral hydrate, the rats were placed in decubitus supine position on the heat pad. The transthoracic echocardiography was carried out using GE ViVid-q ultrasound systems with 3-MHz linear transducer and 2-cm depth two-dimensional imaging (GE Systems, Hayozma, Tirat Carmel, Israel) when pre warmed echo transmission gel was applied to the hairless chest after injection of ISO for 48 h. 

### 4.8. Enzyme-Linked Immunosorbent Assay (ELISA)

The whole blood of the rats was collected with coagulation tube. To obtain the serum, the samples were centrifuged at speed of 2000 rpm for 10 min. The concentration of acrolein-protein adduct, CK-MB and LDH was measured by ELISA (Yi Feixue, Nanjing, China) according to the protocol.

### 4.9. Hochest 33258 to Detect Apoptosis

After the H9c2 cells were seeded in a 6-well plate for 24 h, HT was added at the indicated concentration and incubated for 24 h. Then acrolein was added at the indicated concentration for 12 h. The cells were fixed in 4% paraformaldehyde for 15 min, and washed twice with PBS for 5 min. The cells were washed twice with PBS again after being stained with 500 µL Hoechst 33258 (Beyotime, Shanghai, China) for 5 min. The stained nuclei were observed under an inverted fluorescence microscope (Olympus, Tokyo, Japan).

### 4.10. Flow Cytometry

The apoptosis of H9c2 cells was tested by Annexin-V FITC/PI kit (KeyGEN, Nanjing, China) according to the instructions. H9c2 cells were harvested after the indicated treatment, and then the cells were stained with Annexin-V FITC and PI, which were analyzed by flow cytometry.

### 4.11. Statistical Analyses

Data were presented as the mean ± standard deviation (SD) of at least three independent experiments. Statistical significance of differences between two or more groups was analyzed by student’s two-tailed *t*-test or one-way analysis of variance (ANOVA). GraphPad Prism v5.0 (Graphpad Software, Inc. La Jolla, CA, USA) software was used for statistical analysis. Statistical significance was set at * *p* < 0.05, ** *p* < 0.01 and *** *p* < 0.001.
